# NAFLD fibrosis score is correlated with PCSK9 and improves outcome prediction of PCSK9 in patients with chest pain: a cohort study

**DOI:** 10.1186/s12944-021-01610-w

**Published:** 2022-01-07

**Authors:** Jia Peng, Ming-Ming Liu, Jing-Lu Jin, Ye-Xuan Cao, Yuan-Lin Guo, Na-Qiong Wu, Cheng-Gang Zhu, Qian Dong, Jing Sun, Rui-Xia Xu, Jian-Jun Li

**Affiliations:** Cardiometabolic medicine center, State Key Laboratory of Cardiovascular Diseases, Fu Wai Hospital, National Center for Cardiovascular Diseases, Chinese Academy of Medical Sciences and Peking Union Medical College, No 167 BeiLiShi Road, XiCheng District, Beijing, 100037 China

**Keywords:** Non-alcoholic fatty liver disease fibrosis score, Proprotein convertase subtilisin/kexin type 9, Cardiovascular outcomes

## Abstract

**Background:**

The risk of liver fibrosis in non-alcoholic fatty liver disease (NAFLD) can be easily evaluated by noninvasive scoring systems, of which the NAFLD fibrosis score (NFS) is the most commonly used. Proprotein convertase subtilisin/kexin type 9 (PCSK9), a new predictor of cardiovascular events, has been reported to be associated with cardiovascular outcomes and NAFLD. However, the relationship of NFS with PCSK9 and their prognostic abilities in cardiovascular risks are unknown.

**Methods:**

A total of 2008 hospitalized subjects who had chest pain without lipid-lowering therapy were consecutively included. Baseline clinical data were collected, and the NFS was calculated. The circulating PCSK9 concentration was determined by enzyme immunoassay. The major adverse cardiovascular event (MACE) occurrences were recorded in the follow-up period. Associations of PCSK9 concentration with NFS were examined. All of the participants were categorized into three groups according to NFS levels and were further stratified by PCSK9 tertiles to evaluate the MACEs.

**Results:**

158 (7.87%) MACEs were observed during a mean of 3.2 years of follow-up. NFS levels were independently related to higher PCSK9 levels according to multivariable linear regression analysis. Furthermore, elevated PCSK9 and NFS concentrations were respectively associated with increased MACE incidence in multivariable Cox regression models. When combining NFS status with PCSK9 tertiles as a stratifying factor, patients with intermediate-high NFS and high PCSK9 levels had higher risks of events than those with low NFS and low PCSK9 levels.

**Conclusions:**

This study revealed for the first time that NFS is positively related to PCSK9 and that the combination of NFS and PCSK9 greatly increased the risk of MACEs in patients with chest pain, providing a potential link between NFS and PCSK9 for predicting cardiovascular events.

**Supplementary Information:**

The online version contains supplementary material available at 10.1186/s12944-021-01610-w.

## Introduction

Non-alcoholic fatty liver disease (NAFLD) has been acknowledged as a major public health concern, with an estimated prevalence of 25% in adults, and it shows a continuously increasing trend [1, 2]. Liver fibrosis stage determined by the diagnostic gold standard of liver biopsy is the most important prognostic factor of NAFLD [3]. However, given the risk for complications and high costs, the use of liver biopsy for general screening is limited in the common population [1]. Thus, considering the ever-increasing need for better personalized treatment and prognosis, the risk stratification and continuous evolutionary monitoring of liver disease are increasing [4]. Several noninvasive scoring systems derived from routinely accessible biochemical and clinical parameters have been used as more easily available and safe alternatives to the preliminary estimation of individuals with advanced liver fibrosis, especially for patients without symptoms or a history of liver diseases [5]. The NAFLD fibrosis score (NFS), as the most common noninvasive marker for assessing fibrosis severity in NAFLD, has been demonstrated to be related to liver disease risks and total cause mortality [4]. More interestingly, NFS has been suggested to be linked to coronary artery disease (CAD) incidence and adverse cardiovascular outcomes [6, 7].

 Proprotein convertase subtilisin/kexin type 9 (PCSK9) is mainly synthesized and secreted by liver cells and participates in cholesterol metabolism regulation [8]. Recently, non-lipid-lowering pleiotropic effects of PCSK9 have been proposed, and elevated plasma PCSK9 levels have attracted much attention as a novel marker for cardiovascular events [9, 10]. In addition, the loss of function of the PCSK9 gene has been considered protective against cardiovascular disease, while the gain of functional mutation of PCSK9 exacerbates cardiovascular risks [11]. Strikingly, there is a clear relationship between the functional gain of PCSK9 and liver damage in NAFLD [12]. Moreover, it has been reported that circulating PCSK9 concentration is associated with pathological liver damage [13]. However, the relationship between PCSK9 and general noninvasive scores has not been evaluated, and it is unknown whether NFS can enhance the predictive power of PCSK9 for major adverse cardiovascular events (MACEs).

Hence, the current study, aimed to assess the correlation of NFS with PCSK9 and the prognostic value for MACEs according to the combination of NFS and PCSK9 in chest pain patients without lipid-lowering therapies before hospitalization.

## Methods

### Study population

This study was approved by the local ethical review board (Fu Wai Hospital and National Center for Cardiovascular Diseases, Beijing, China) and strictly implemented according to the Declaration of Helsinki. Each registered patient signed an informed written consent form.

**Fig. 1:**
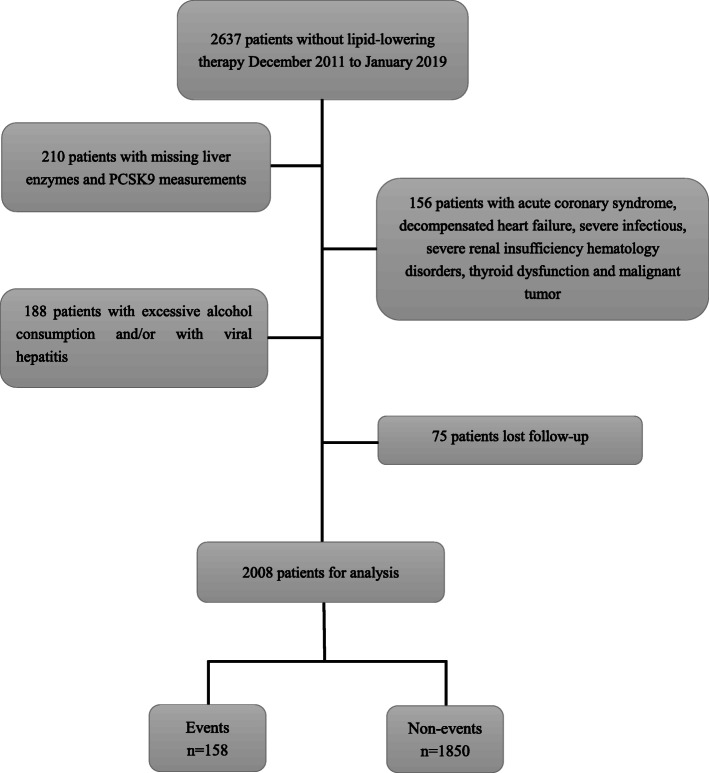
Flowchart of the study. *PCSK9* proprotein convertase subtilisin/kexin type 9

A total of 2637 chest pain patients without lipid-lowering treatment within 3 months of hospitalization from December 2011 to January 2019 were consecutively recruited in this study. The study flowchart is shown in Fig. 1. Patients without PCSK9 measurements and liver enzyme data and those who had excessive alcohol consumption and viral hepatitis B and C were excluded. Excessive alcohol consumption is defined as more than 21 drinks per week for men and more than 14 drinks per week for women, wherein one drink has 12 g of alcohol, as previously described [14, 15]. In addition, patients with acute coronary syndrome, acute and chronic decompensated heart failure, severe renal dysfunction, thyroid insufficiency, hematological disorders, severe infections, and malignant tumors were excluded. Thus, 2083 eligible patients were enrolled in the present study and followed up for MACEs. Subsequently, 75 patients were lost to follow-up. Finally, the data from 2008 patients who did not take lipid-lowering treatments before hospitalization were analyzed. All of the subjects received precise therapy, including lipid-lowering treatment, according to their condition after discharge.

### Follow-up

The follow-up of all individuals was conducted by well-trained investigators by telephone every 6 months. Clinical data from each participant and the aim of the current study were blind. The present study continued until December 31, 2018. MACEs included hospitalization for unstable angina, ischemic stroke, nonfatal myocardial infarction, cardiovascular death, and coronary revascularization.

### Measurements and biochemical analysis

Baseline characteristics of each participant, including medical history, medication use, demographic data, and lifestyle characteristics were collected.

After hospitalization and fasting for 12 h overnight, the blood samples from each patient were collected from the cubital vein. The circulating PCSK9 concentration was determined by a high-sensitivity and quantitative ELISA (R&D, California, USA) according to a previous study [16]. The circulating PCSK9 concentration was measured twice in every subject, and the average value of the two determinations was considered as the final concentration. Routine blood lipid parameters and fasting plasma glucose (FPG) were measured using Hitachi 7150 (Hitachi, Tokyo, Japan). Serum liver enzymes were determined using the same standard laboratory methods at the Fu Wai Hospital. Hemoglobin A1c (HbA1c) was detected by HLC-723G8 (Tosoh, Tokyo, Japan).

### NFS

NFS was calculated by the following specific formula: = − 1.675 + 0.037 × age (years) + 0.094 × body mass index (BMI) (kg/m^2^) + 1.130 × prediabetes or diabetes (yes: 1, no: 0) + 0.990 × (aminotransferase [AST] [IU/L]/ aminotransferase [ALT] [IU/L]) − 0.013 × platelet count (10^9^/L) − 0.660 × albumin (g/dL) [17]. Patients were divided into low, intermediate, and high NFS groups according to NFS cutoff values (− 1.455 and 0.676), reflecting the risks of liver fibrosis [17]. Because of the lower incidence of MACEs in this entire population, the intermediate and high NFS groups were merged into one group to evaluate MACEs. To further examine the combined impacts of NFS and PCSK9 on MACEs, all patients were divided into 6 subgroups by NFS status and PCSK9 tertiles (low PCSK9 plus low NFS group as the reference group, low PCSK9 plus intermediate-high NFS group, intermediate PCSK9 plus low NFS group, intermediate PCSK9 plus intermediate-high NFS group, high PCSK9 plus low NFS group, and high PCSK9 plus intermediate-high NFS group).

### Statistical analysis

Data are presented as the means ± standard deviation (SD) or medians (25th–75th percentile) for continuous indices and as numbers (percentage) for categorical indices. The distribution pattern of variables was tested by Kolmogorov-Smirnov analysis. Significant differences in parameters were assessed by Student’s *t* test, the Mann-Whitney *U* test, or the *λ*^2^ tests between groups where appropriate. The relationship of NFS with PCSK9 was examined by linear regression analyses (univariable and multivariable). Furthermore, event-free survival rates were examined by Kaplan-Meier curve analysis with the log-rank test among groups. Hazard ratios (HRs) with 95% confidence intervals (CIs) for MACEs in different groups were assessed by Cox regression analyses. Sensitivity analysis with regard to drinking was performed for the association of PCSK9 and NFS with MACEs, which was forced into multivariate models together with PCSK9 or NFS. A *P* value < 0.05 indicated a statistically significant difference. SPSS software, version 25.0, was applied to perform all statistical analyses.

## Results

### Baseline characteristics

The average age of the entire population was 55.5 ± 10.9 years old, and 1215 (60.6%) subjects were male. The interquartile plasma levels of PCSK9 ranged from 190.57 to 272.29 ng/mL, and the median level of PCSK9 was 228.56 ng/mL. The NFS levels were in the range of − 6.26 to 4.27. The baseline characteristics stratified according to the occurrence of events during the follow-up period are presented in Table 1. Participants with events were older and had a higher occurrence of CADand diabetes; and a lower percentage of family history of CAD; and higher gamma-glutamyltransferase (GGT) levels; and a higher usage of prescription medication (including antiplatelet and antihypertensive drugs) than those without events (all *P* <  0.05). Importantly, higher NFS and PCSK9 concentrations were observed in individuals with events than in those without (all *P* <  0.05), while the proportions of male sex, hypertension, blood pressure, smoking and drinking status; BMI, and triglyceride, total cholesterol (TC), low-density lipoprotein cholesterol (LDL-C), platelet count, AST, ALT, albumin, FPG, HbA1c levels were not significantly different in patients with and without (all *P* >  0.05).

**Table 1:** Baseline characteristics in study patients with and without events

Variables	Total	Events	Non-events	***P***
	***n*** = 2008	***n*** = 158	***n*** = 1850	
**Clinical data**				
Age (years)	55.5 ± 10.9	57.5 ± 9.6	55.3 ± 11.0	**0.017**
Male sex, n(%)	1215 (60.6)	101 (63.9)	1114 (60.2)	0.360
BMI (kg/m^2^)	25.7 ± 3.4	26.0 ± 3.5	25.6 ± 3.4	0.185
CAD, n(%)	1179 (58.7)	121 (76.6)	1058 (57.2)	**< 0.0001**
DM, n(%)	471 (23.5)	54 (34.2)	417 (22.5)	**0.001**
Hypertension, n(%)	1229 (61.2)	107 (67.7)	1122 (60.6)	0.080
Family history of CAD, n(%)	440 (21.9)	22 (13.9)	418 (22.6)	**0.011**
Smoking, n(%)	683 (34.0)	56 (35.4)	627 (33.9)	0.693
Drinking, n(%)	417 (20.8)	35 (22.2)	382 (20.6)	0.655
SBP (mmHg)	127.6 ± 18.5	130 ± 16.9	127.4 ± 18.7	0.099
DBP (mmHg)	79.5 ± 11.2	79.8 ± 11.8	79.5 ± 11.1	0.710
**Laboratory parameters**				
Platelet (10^9^/L)	216.0 ± 53.6	208.7 ± 51.1	216.6 ± 53.8	0.075
ALT (IU/L)	20 (14,28)	21 (14,29)	20 (14,28)	0.472
AST (IU/L)	18 (15,22)	18 (14.75,22)	18 (15,22)	0.806
GGT (IU/L)	25 (18,39)	30 (21,44.25)	25 (18,39)	**< 0.0001**
ALB (g/L)	42.5 ± 4.3	41.9 ± 3.8	42.6 ± 4.3	0.060
TC (mmol/L)	4.80 ± 1.01	5.00 ± 0.94	4.80 ± 1.01	0.095
TG (mmol/L)	1.60 (1.15,2.31)	1.60 (1.24,2.36)	1.60 (1.14,2.30)	0.541
HDL-C (mmol/L)	1.11 ± 0.39	1.13 ± 0.35	1.11 ± 0.39	0.539
LDL-C (mmol/L)	3.13 ± 0.91	3.24 ± 0.81	3.12 ± 0.92	0.128
Glucose (mmol/L)	5.66 ± 1.96	5.91 ± 2.61	5.64 ± 1.90	0.094
HbA1C (%)	6.05 ± 1.88	6.3 ± 1.11	6.03 ± 1.93	0.081
PCSK9 (ng/mL)	228.56 (190.57,272.29)	245.23 (204.53,292.15)	227.58 (189.93,270.97)	**0.002**
NFS	−1.09(−1.98,-0.27)	−0.77(−1.53,0.05)	−1.12(−2.00,-0.29)	**< 0.0001**
**Medication**				
Antiplatelet drugs, n(%)	595 (29.6)	65 (41.4)	530 (28.6)	**0.001**
Antihypertensive drugs, n(%)	694 (34.6)	67 (42.4)	627 (33.9)	**0.031**

*PCSK9* proprotein convertase subtilisin/kexin type 9, *BMI* body mass index, *CAD* coronary artery disease, *DM* diabetes mellitus, *SBP* systolic blood pressure, *DBP* diastolic blood pressure, *ALT* alanine aminotransferase, *AST* aspartate aminotransferase, *GGT* gamma-glutamyl transpeptidase, *ALB* albumin, *TC* total cholesterol, *TG* triglyceride, *HDL-C* high-density lipoprotein cholesterol, *LDL-C* low-density lipoprotein cholesterol, *HbA1C* hemoglobin A1C, *NFS* non-alcoholic fatty liver disease fibrosis score, *P* <  0.05 suggests significant difference

In addition, the baseline characteristics of the study population according to NFS status are shown in Table 2. There was an ascending gradient with regard to the proportions of women, CAD and diabetes; the baseline levels of age, BMI, systolic blood pressure, HbA1c and PCSK9; and the usage of antihypertensive drugs across NFS status (all *P* <  0.05). Furthermore, a descending gradient was observed in the percentages of smoking and family history of CAD, platelet counts, ALT, GGT, albumin and TC levels among the three NFS groups (all *P* <  0.05). However, no significant differences in the levels of diastolic blood pressure, high-density lipoprotein cholesterol (HDL-C), LDL-C, and AST and the proportion of alcohol consumption were observed among the three NFS groups (all *P* >  0.05).

**Table 2:** Baseline characteristics of different groups according to NFS status

Variables		NFS levels			***P***
		Low	Intermediate	High	
	Overall	< −1.455	−1.455-0.676	> 0.676	
	***n*** = 2008	***n*** = 798	***n*** = 1072	***n*** = 138	
**Clinical data**					
Age (years)	55.5 ± 10.9	49.2 ± 10.1	58.7 ± 8.9	66.8 ± 8.7	**< 0.0001**
Male sex, n(%)	1215 (60.6)	506 (63.4)	636 (59.3)	73 (52.9)	**0.034**
BMI (kg/m^2^)	25.7 ± 3.4	25 ± 3.2	26 ± 3.3	26.6 ± 4.1	**< 0.0001**
CAD, n(%)	1179 (58.7)	401 (50.3)	681 (63.5)	97 (70.3)	**< 0.0001**
DM, n(%)	471 (23.5)	93 (11.7)	327 (30.5)	51 (37.0)	**< 0.0001**
Hypertension, n(%)	1229 (61.2)	426 (53.4)	703 (65.6)	100 (72.5)	**< 0.0001**
Family history of CAD, n(%)	440 (21.9)	195 (24.4)	235 (21.9)	10 (7.2)	**< 0.0001**
Smoking, n(%)	683 (34.0)	303 (38.0)	351 (32.7)	29 (21.0)	**< 0.0001**
Drinking, n(%)	417 (20.8)	185 (23.2)	210 (19.6)	22 (15.9)	0.058
SBP (mmHg)	127.6 ± 18.5	125.2 ± 18	128.7 ± 18.7	132.7 ± 18.5	**< 0.0001**
DBP (mmHg)	79.5 ± 11.2	79.9 ± 11.7	79.4 ± 10.9	77.8 ± 10.4	0.117
**Laboratory parameters**					
Platelet (10^9^/L)	216.0 ± 53.6	247.36 ± 52.84	200.11 ± 40.91	157.41 ± 40.38	**< 0.0001**
ALT (IU/L)	20 (14,28)	22 (16,32)	19 (14,27.75)	14 (10,20)	**< 0.0001**
AST (IU/L)	18 (15,22)	18 (15,22)	18 (15,21)	18.5 (15,24)	0.375
GGT (IU/L)	25 (18,39)	26.5 (18,43)	24 (18,37)	22 (16,33)	**< 0.0001**
ALB (g/L)	42.5 ± 4.3	44.0 ± 4.4	41.9 ± 3.8	39.0 ± 4.1	**< 0.0001**
TC (mmol/L)	4.8 ± 1.01	4.90 ± 1.08	4.90 ± 0.95	4.60 ± 0.96	**0.021**
TG (mmol/L)	1.6 (1.15,2.31)	1.61 (1.15,2.38)	1.62 (1.18,2.31)	1.42 (1.06,1.98)	**0.018**
HDL-C (mmol/L)	1.11 ± 0.39	1.10 ± 0.43	1.12 ± 0.37	1.06 ± 0.30	0.109
LDL-C (mmol/L)	3.13 ± 0.91	3.14 ± 1.00	3.14 ± 0.85	2.98 ± 0.79	0.079
Glucose (mmol/L)	5.66 ± 1.96	5.00 ± 1.26	5.91 ± 2.36	5.73 ± 1.57	**< 0.0001**
HbA1C (%)	6.05 ± 1.88	5.75 ± 2.02	6.24 ± 1.82	6.31 ± 0.92	**< 0.0001**
PCSK9(ng/mL)	228.56 (190.57,272.29)	224.72 (184.54,265.17)	230.13 (193.44,275.58)	244.28 (194.15,284.31)	**0.005**
**Medication**					
Antiplatelet drugs, n(%)	595 (29.6)	189 (23.7)	361 (33.7)	45 (32.6)	**< 0.0001**
Antihypertensive drugs, n(%)	694 (34.6)	222 (27.8)	414 (38.6)	58 (42.0)	**< 0.0001**

*NFS* non-alcoholic fatty liver disease fibrosis score, *PCSK9* proprotein convertase subtilisin/kexin type 9, *BMI* body mass index, *CAD* coronary artery disease, *DM* diabetes mellitus, *SBP* systolic blood pressure, *DBP* diastolic blood pressure, *ALT* alanine aminotransferase, *AST* aspartate aminotransferase, *GGT* gamma-glutamyl transpeptidase, *ALB* albumin, *TC* total cholesterol, *TG* triglyceride, *HDL-C* high-density lipoprotein cholesterol, *LDL-C* low-density lipoprotein cholesterol, *HbA1C* hemoglobin A1C, *P* < 0.05 suggests significant difference

### Relationship between NFS and PCSK9

A significant, positive correlation was found between plasma levels of PCSK9 and NFS in the univariable linear regression model (β = 0.077, *P* = 0.001, Table 3). These associations were further evaluated after adjusting for traditional risk factors by stepwise multivariate linear regression analysis. After adjusting for gender, the results indicated that a positive association of PCSK9 levels with NFS remained significant (β = 0.066, *P* = 0.003, Table 3). Even after further adding BMI, family history of CAD, hypertension, smoking status, drinking status, triglycerides, LDL-C, HDL-C, FPG, and HbA1c into the multivariate linear regression model, the positive relation of PCSK9 levels with NFS remained significantly different (β = 0.073, *P* = 0.001, Table 3).

**Table 3:** Liner regression analysis between NFS and PCSK9

	Independent variable NFS	
	Standard coefficient β	***P***
**Model 1**	0.077	**0.001**
**Model 2**	0.066	**0.003**
**Model 3**	0.065	**0.003**
**Model 4**	0.069	**0.002**
**Model 5**	0.069	**0.002**
**Model 6**	0.073	**0.001**

PCSK9 is the dependent variable. NFS, non-alcoholic fatty liver disease fibrosis score

Model 1 was unadjusted model

Model 2 was adjusted for gender

Model 3 was adjusted for model 2 covariates plus body mass index

Model 4 was adjusted for model 3 covariates plus smoking and drinking

Model 5 was adjusted for model 4 covariates plus hypertension and family history of coronary artery disease

Model 6 was adjusted for model 5 covariates plus triglyceride, high-density lipoprotein cholesterol, low-density lipoprotein cholesterol, fasting plasma glucose and hemoglobin A1c

*P* < 0.05 suggests significant difference

### Relationship between NFS, PCSK9, and outcomes

**Fig. 2:**
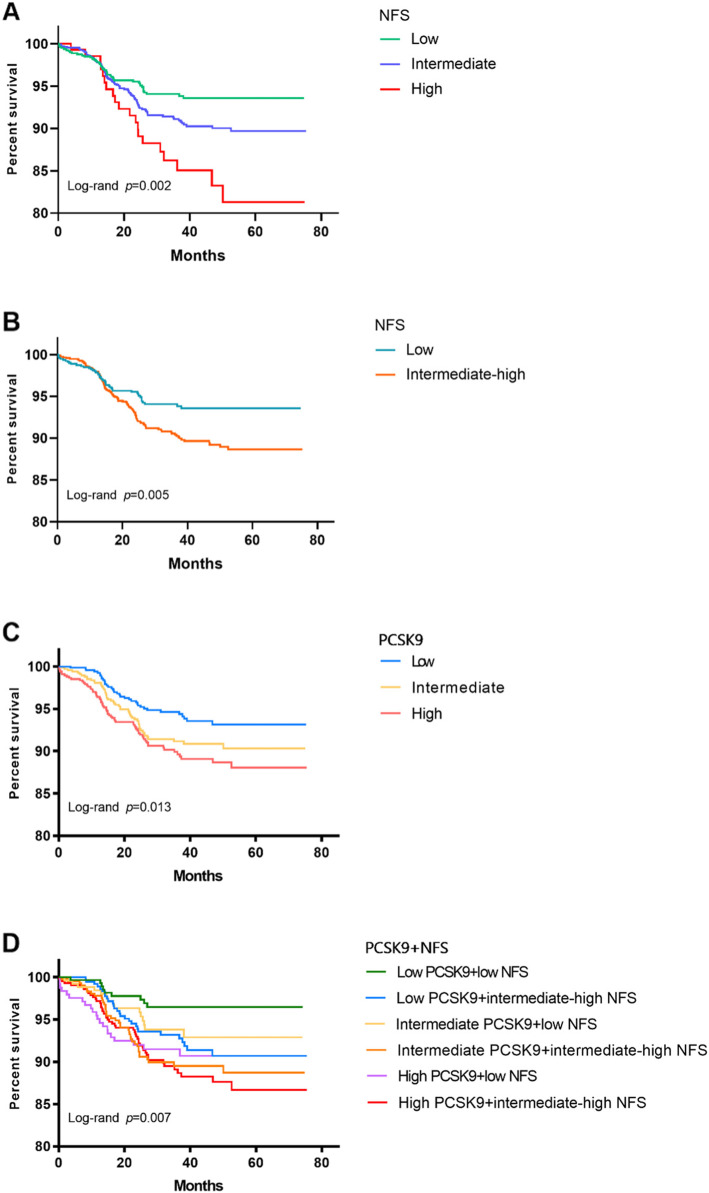
Kaplan-Meier analysis for (**A** and **B)** the categories of NFS, (**C**) the categories of PCSK9, and (**D**) the categories of combination with NFS and PCSK9. *NFS* non-alcoholic fatty liver disease fibrosis score, *PCSK9* proprotein convertase subtilisin/kexin type 9

For 6389 person-years, 158 (7.87%) MACEs were recorded (92 experienced hospitalization because of unstable angina pectoris, 41 underwent unplanned coronary revascularization, 2 suffered nonfatal MI, 16 had ischemic stroke, and 7 died). The event-free survival rate for MACEs was the lowest in the high NFS group among the three groups (*P* for all comparisons = 0.002, Fig. 2A) according to the Kaplan-Meier curve. Similarly, as shown in Fig. 2B, a higher risk of future events was observed in individuals with intermediate-high NFS levels than in patients with low NFS levels (*P* <  0.05). Furthermore, the highest event rate for MACEs was appeared in the high PCSK9 group among the three groups (*P* for all comparisons = 0.013, Fig. 2C). However, no statistically significant difference was found between the intermediate and high PCSK9 groups or between the low and intermediate groups (*P* > 0.05, Fig. 2C). Next, all individuals were categorized into six subgroups on the basis of NFS status and PCSK9 tertiles. The high PCSK9 plus intermediate-high NFS group was more likely to show an elevated incidence of MACEs than the reference groups (*P* <  0.05), while there was no significant difference between the low PCSK9 plus intermediate-high NFS group and the reference group (all *P* > 0.05, Fig. 2D).

Significantly gradual correlations were found from the low, intermediate, and high groups based on NFS and PCSK9 status (Table 4). In multivariate Cox regression analysis, participants with high NFS levels or high PCSK9 levels had 1.935-fold and 1.683-fold higher risks of subsequent events than subjects in the low NFS or low PCSK9 groups, respectively (all *P* <  0.05). Moreover, after adjustment for traditional risk factors, per SD increases in NFS and PCSK9 resulted in 22.2 and 22.4% increases in the risks of subsequent MACEs, respectively (all *P* < 0.05, Table 4). Considering the underlying impacts of drinking on PCSK9 and liver function [18, 19], sensitivity analyses were performed, and the results were persistent when drinking was forced into models with PCSK9 or NFS (all *P* < 0.05, Supplementary Table S1). Notably, the combined impact of NFS and PCSK9 on cardiovascular outcomes was further estimated, and the results are shown in Table 4. After adjustment for confounding factors, the multivariable-adjusted HRs (95% CI) among all patients in the intermediate PCSK9 plus intermediate-high NFS group, in the high PCSK9 plus low NFS group, and in the high PCSK9 plus intermediate-high NFS group were 2.369(1.134–4.951), 2.689(1.219–5.934), and 2.693(1.295–5.599) times higher than those in the reference group for MACEs, respectively. At the same time, the interaction of NFS and PCSK9 with cardiovascular events was not significantly different (*P* for interaction = 0.055).

**Table 4:** Relation of NFS and PCSK9 levels with MACEs in all patients

	Events/subjects	Model 1		Model 2	
		HR(95%CI)	***p***	HR(95%CI)	***p***
**NFS**					
per 1-SD increase	158/2008	1.353 (1.154,1.586)	**< 0.0001**	1.222 (1.027,1.453)	**0.024**
Low	45/798	1.0		1.0	
Intermediate	93/1072	1.516 (1.062,2.164)	**0.022**	1.245 (0.859,1.804)	0.248
High	20/138	2.561 (1.511,4.341)	**< 0.0001**	1.935 (1.107,3.381)	**0.020**
**PCSK9**					
per 1-SD increase	158/2008	1.277 (1.097,1.486)	**0.002**	1.224 (1.047,1.431)	**0.011**
Low	37/669	1.0		1.0	
Intermediate	54/670	1.499 (0.985,2.281)	0.059	1.366 (0.894,2.087)	0.149
High	67/669	1.850 (1.229,2.784)	**0.003**	1.683 (1.109,2.554)	**0.014**
**PCSK9 + NFS**	158/2008				
Low PCSK9 + low NFS	9/297	1.0		1.0	
Low PCSK9 + intermediate-high NFS	28/372	2.399 (1.132,5.085)	**0.022**	1.948 (0.914,4.151)	0.084
Intermediate PCSK9 + low NFS	15/258	1.986 (0.869,4.540)	0.104	1.802 (0.783,4.147)	0.166
Intermediate PCSK9 + intermediate-high NFS	39/412	3.237 (1.568,6.685)	**0.001**	2.369 (1.134,4.951)	**0.022**
High PCSK9 + low NFS	21/243	2.966 (1.357,6.486)	**0.006**	2.689 (1.219,5.934)	**0.014**
High PCSK9 + intermediate-high NFS	46/426	3.677 (1.792,7.546)	**< 0.0001**	2.693 (1.295,5.599)	**0.008**

*NFS* non-alcoholic fatty liver disease fibrosis score, *PCSK9* proprotein convertase subtilisin/ kexin type 9, *MACEs* major adverse cardiovascular events, *HR* hazard ratio, *CI* confidence interval. Model 1 was adjusted for age and gender, except NFS (only adjusted for gender). Model 2 was adjusted for model 2 covariates plus body mass index, hypertension, family history of coronary artery disease, coronary artery disease, diabetes, smoking, drinking, triglyceride, high-density lipoprotein cholesterol, low-density lipoprotein cholesterol, fasting plasma glucose, hemoglobin A1c, antiplatelet drugs, antihypertensive drugs. *p* < 0.05 suggests significant difference

In addition, a sex-based comparison of the associations of PCSK9 and NFS with MACEs was explored in the present study. As presented in Table 5, SD changes in NFS and PCSK9 were related to 52.8 and 34.4% elevated risks of cardiovascular events in men, respectively (all *P* < 0.05). Multivariable Cox regression analysis according to NFS and PCSK9 levels showed a 3.377-fold (95% CI: 1.394–8.179) higher prevalence in men in the high PCSK9 plus intermediate-high NFS group than in the low PCSK9 plus low NFS group (*P* < 0.05). However, the significant relationship of NFS, PCSK9, and the combination of NFS and PCSK9 with MACEs disappeared in women (all *P* > 0.05).

**Table 5:** Cox regression analysis of PCSK9, NFS and the combination of NFS and PCSK9 with events in male and female

Variables		Male			Female	
		Multivariable model			Multivariable model	
	Events/ Subjects	OR(95%CI)	***P***	Events/ Subjects	OR(95%CI)	***P***
NFS per 1-SD increase	101/1215	1.528 (1.147,2.035)	**0.004**	57/793	1.033 (0.739,1.446)	0.848
PCSK9 per 1-SD increase	101/1215	1.344 (1.102,1.64)	**0.004**	57/793	1.042 (0.81,1.341)	0.747
PCSK9 + NFS						
Low PCSK9 + low NFS	7/210	1.0		2/87	1.0	
Low PCSK9 + intermediate-high NFS	20/265	1.907 (0.774,4.696)	0.161	8/107	2.32 (0.465,11.588)	0.310
Intermediate PCSK9 + low NFS	10/162	1.729 (0.65,4.603)	0.273	5/96	2.216 (0.419,11.721)	0.350
Intermediate PCSK9 + intermediate-high NFS	26/253	2.382 (0.985,5.764)	0.054	13/159	2.511 (0.524,12.024)	0.250
High PCSK9 + low NFS	11/134	2.236 (0.855,5.846)	0.101	10/109	3.361 (0.708,15.95)	0.130
High PCSK9 + intermediate-high NFS	27/191	3.377 (1.394,8.179)	**0.007**	19/235	2.178 (0.468,10.131)	0.320

*PCSK9* proprotein convertase subtilisin/ kexin type 9, *NFS* non-alcoholic fatty liver disease fibrosis score, *MACEs* major adverse cardiovascular events, *HR* hazard ratio, *CI* confidence interval. Multivariable model was adjusted for body mass index, hypertension, family history of coronary artery disease, coronary artery disease, diabetes, smoking, drinking, triglyceride, high-density lipoprotein cholesterol, low-density lipoprotein cholesterol, fasting plasma glucose, hemoglobin A1c, antiplatelet drugs, antihypertensive drugs. *P* < 0.05 suggests significant difference

## Discussion

This study examined the relationship of NFS with PCSK9 and determined the ability of combining NFS and PCSK9 to predict MACEs in patients with chest pain who have not received any lipid-lowering medications before admission. First, baseline levels of NFS were significantly and positively correlated with plasma PCSK9 concentration. Furthermore, multivariate Cox regression analysis indicated that high levels of NFS and PCSK9 were related to MACEs. When stratified by the combination of NFS status and PCSK9 tertiles, both patients in the high PCSK9 plus any NFS status groups and patients in the intermediate-high NFS plus intermediate PCSK9 group showed higher risks for MACEs than individuals in the reference group.

Given the functional diversity of PCSK9, it has been considered to be a new prognostic factor for cardiovascular events in high-risk populations including CAD and family hypercholesterolemia [20, 21]. Furthermore, PCSK9 has been proposed to be positively related to cardiovascular Framingham Risk Score in obese subjects [22]. Additionally, a cohort study with a 2-year follow-up found that higher PCSK9 levels predicted cardiovascular outcomes in 504 stable CAD patients on statin therapy [23], while, a meta-analysis containing eight cohort studies revealed that the circulating concentration of PCSK9 was positively associated with an elevated risk of total adverse cardiovascular outcome [24]. In contrast, a negative relation of PCSK9 to cardiovascular risks has been reported in a cohort study including 1527 men without vascular disease and in a nested case-control evaluation in 358 cases and 358 controls [25, 26]. Therefore, the ability of PCSK9 to predict risks must still be investigated. It is known that PCSK9 is mainly secreted by liver cells and is involved in lipid metabolism [27]. Moreover, a recent study found that hepatic reentry of plasma PCSK9 triggered the sensing loop regulating PCSK9 and low-density lipoprotein secretion [28], suggesting that liver dysfunction could change the circulating PCSK9 concentration and influence its function. Whether liver damage, especially liver fibrosis, has an impact on the predictive ability of PCSK9 for adverse cardiovascular outcomes is worth exploring.

The NFS is the most common simple scoring system, and it consists of seven regular clinical and laboratory indices, including age, diabetes status, platelet counts, albumin, BMI, ALT, and AST, to predict advanced liver fibrosis and reflect the progression of liver injury in patients with NAFLD [17]. Currently, the clinical usage of the NFS has been extended to detect liver complications and death, not only in ultrasonography-diagnosed NAFLD individuals but also in the general population [29, 30]. Notably, NFS appeared to be the best prognostic predictor of patients at risk compared with other simple noninvasive scoring systems during a long-term follow-up [31]. Furthermore, the NFS has served as a cardiovascular risk predictor since seven parameters included in the NFS are associated with cardiometabolic risks. In addition, a prior cross-sectional study revealed that NFS was independently related to the complexity of CAD [32]. Likewise, a prospective and observational cohort study including 3263 CAD patients with a 7.56-year follow-up period found an obvious association between NFS and enhanced risks of cardiovascular and all-cause mortality [6]. Nevertheless, there is still little research on the prognostic value of NFS for cardiovascular events, particularly in different populations.

In the present study, a positive relationship between NFS and PCSK9 was found, which was supported by Lebeau’s team who used hepatocyte-specific PCSK9-knockout mice and showed that PCSK9 blockade or deficiency conferred resistance to liver steatosis to repair hepatic damage [33]. Moreover, Lebeau et al. [34] demonstrated that diet-induced hepatic steatosis elevated circulating PCSK9 concentrations as a result of de novo expression in mice by abrogating hepatic low-density lipoprotein receptor expression. In addition, a clinical study enrolled 201 patients undergoing liver biopsy for suspected NAFLD, examined the relation of PCSK9 with liver damage and showed that circulating concentration of PCSK9 was significantly related to liver steatosis grade (*P* = 0.012) [13]. Considered together, the above studies indicated that PCSK9 might have a critical impact on the progression of NAFLD, consistent with the present results.

Another important finding of this study was that NFS and PCSK9 were independently associated with MACEs. When NFS and PCSK9 status were combined as a stratification factor in the multivariate Cox regression model, NFS promoted the protective ability of PCSK9 on MACEs in chest pain patients. An increasing number of clinical studies have indicated that NAFLD could precede the development of atherosclerotic cardiovascular disease and that NAFLD was significantly correlated with all-cause death risks in the long term, mainly deriving from cardiovascular complications [35, 36]. More importantly, liver fibrosis is the irreversible progression of NAFLD, and fibrosis stage is the only critical histological characteristic independently related to poor prognosis [37]. Furthermore, it is noteworthy that PCSK9 has an adverse effect on NAFLD development and is correlated with cardiometabolic factors or metabolic syndrome [38]. In addition, NFS is calculated using multiple cardiometabolic and cardiovascular risk-related parameters and reflects liver fibrosis in NAFLD [6, 17]. This evidence supported that the underlying connection between NFS and PCSK9 could be bridged by NAFLD.

Alcohol consumption or excessive alcohol consumption has recently been proposed to be related to liver injury with increased liver function enzymes and epigenetic regulation of PCSK9, which is involved in cardiovascular risks [18, 19]. Moreover, PCSK9 inhibition with alirocumab treatment could alleviate alcohol-induced steatohepatitis by attenuating alcohol-induced hepatocellular injury, hepatic inflammation, and neutrophil infiltration [39]. Thus, individuals with excessive alcohol consumption were excluded to avoid the bias of results, and sensitivity analyses showed that PCSK9 and NFS were associated with cardiovascular outcomes after adjusting for drinking, rendering the findings of the present study more reliable.

In addition, sex differences have reportedly appeared in PCSK9 levels and cardiovascular diseases [40]. Consistent with previous studies, higher PCSK9 and NFS levels were observed in women than in men [PCSK9: 243.7(203.98, 289.21) vs. 220.29(181.85,259.42) ng/mL, *P* <  0.001; NFS: − 1.02(− 1.91, − 0.18) vs. − 1.15 (− 2.014, − 0.33), *P* = 0.018] in the current study. Sex differences in predicting cardiovascular risks based on NFS and PCSK9, as well as the combination of the two indicators, are worth investigating. As a result, NFS and PCSK9 levels were related to cardiovascular outcomes in men but not in women. Similarly, an additive effect of NFS and PCSK9 on cardiovascular events was found in men. Hormones such as estrogen seem to play a critical role in such a phenomenon [41]. More studies will be conducted to identify potential sex-related differences in the association of PCSK9 and NFS levels with cardiovascular risk.

### Comparisons with other studies and what does the current work add to the existing knowledge

The association between PCSK9 and liver damage including liver fibrosis, in subjects with NAFLD or with liver cirrhosis has been explored recently [42, 43]. However, there are no data regarding to the relationship between PCS9 and noninvasive liver fibrosis score systems in patients suspected of having CAD. Furthermore, the effect of the combination of PCSK9 and noninvasive liver fibrosis scoring systems on cardiovascular risks lacks evidence. This study provided new insights into the relationships among PCSK9, liver fibrosis, and cardiovascular risks in patients suspected of having CAD, further raising the question of whether PCSK9 inhibitors may have considerable cardiovascular benefits in patients with liver fibrosis. Further studies should be conducted to investigate and resolve this uncertainty.

### Study strengths and limitations

This study included a relatively large Chinese population that was not administered lipid-lowering treatment before admission to evaluate for the first time the relationships among PCSK9, noninvasive liver fibrosis score, and cardiovascular events, providing novel information in the field of cardiovascular and liver disease. However, the limitations of this study should be mentioned. First, this study only calculated and collected the baseline levels of NFS and PCSK9 and could not investigate their changes or their influence on MACE prediction during follow-up. Second, alcohol consumption was self-reported, as in most studies, and subjects who had excessive alcohol consumption were excluded. Meanwhile, the multivariate analyses were adjusted for drinking status to avoid bias. Third, the present study sample comprised hospitalized Chinese patients from a single center, which had an effect on the generalizability of the results. These findings require observation and replication in other populations. Finally, as the one of nature of the observational prospective study, the causal relationship between NFS and PCSK9, as well as cardiovascular risks, has not been clearly determined. Importantly, these findings were subject to unmeasured confounding factors. Therefore, additional studies need be conducted to confirm the relationships among PCSK9, NFS, and cardiovascular outcomes.

## Conclusions

In conclusion, this study firstly found a positive correlation of NFS with PCSK9. More importantly, elevated NFS and PCSK9 could better predict adverse cardiovascular events in patients with angina-like chest pain, indicating that NFS might improve the ability of PCSK9 to predict outcomes. Therefore, prospective studies including participants of different races and populations and the underlying mechanisms of PCSK9 and liver fibrosis are required to validate the association between PCSK9 and liver fibrosis.

## Supplementary Information

**Additional file 1.**

## Data Availability

The datasets used and analyzed during the current study are available from the corresponding author on reasonable request.

## Abbreviations

NAFLD: Non-alcoholic fatty liver disease
NFS: Non-alcoholic fatty liver disease fibrosis score
CAD: Coronary artery disease
PCSK9: Proprotein convertase subtilisin/kexin type 9
MACEs: Major adverse cardiovascular events
TC: Total cholesterol
LDL-C: Low-density lipoprotein cholesterol
FPG: Fasting plasma glucose
HbA1c: Hemoglobin A1c
HDL-C: High-density lipoprotein cholesterol
ALT: Alanine aminotransferase
AST: Aspartate aminotransferase
GGT: Gamma-glutamyl transferase
BMI: Body mass index
SD: Standard deviation
HR: Hazard ratio
CI: Confidence interval
