# Supplementary material for: NAFLD fibrosis score is correlated with PCSK9 and improves outcome prediction of PCSK9 in patients with chest pain: a cohort study
Source: Lipids Health Dis. 2022 Jan 7;21:3. doi: 10.1186/s12944-021-01610-w (PMC8742334; doi:10.1186/s12944-021-01610-w)
Supplement: Supplementary file 1 — Additional file 1. [file 12944_2021_1610_MOESM1_ESM.docx]

**Supplementary**

**Table S1.** **Sensitivity analysis of the association of PCSK9, NFS with MACEs after adjustment for drinking.**

|  | **Multivariable analysis** | |
| --- | --- | --- |
| **Variables** | **HR(95%CI)** | ***P*** |
| PCSK9 per 1-SD increase | 1.275(1.099,1.478) | **0.001** |
| NFS per 1-SD increase | 1.352(1.154,1.585) | **<0.001** |

PCSK9, proprotein convertase subtilisin/ kexin type 9; NFS, non-alcoholic fatty liver disease fibrosis score; MACEs, major adverse cardiovascular events; HR, hazard ratio; CI, confidence interval, *P* < 0.05 suggests significant difference.
